# Artesunate-Amodiaquine and Artemether-Lumefantrine Therapies and Selection of *Pfcrt* and *Pfmdr1* Alleles in Nanoro, Burkina Faso

**DOI:** 10.1371/journal.pone.0151565

**Published:** 2016-03-31

**Authors:** Paul Sondo, Karim Derra, Seydou Diallo Nakanabo, Zekiba Tarnagda, Adama Kazienga, Odile Zampa, Innocent Valéa, Hermann Sorgho, Ellis Owusu-Dabo, Jean-Bosco Ouédraogo, Tinga Robert Guiguemdé, Halidou Tinto

**Affiliations:** 1 IRSS / Clinical Research Unit of Nanoro (CRUN), Nanoro, Burkina Faso; 2 Centre Muraz of Bobo-Dioulasso, Bobo-Dioulasso, Burkina Faso; 3 Kumasi Center for Collaborative Research in Tropical Medicine (KCCR), Kumasi, Ghana; Mahidol Oxford Tropical Medicine Research Unit, THAILAND

## Abstract

The adoption of Artemisinin based combination therapies (ACT) constitutes a basic strategy for malaria control in sub-Saharan Africa. Moreover, since cases of ACT resistance have been reported in South-East Asia, the need to understand *P*. *falciparum* resistance mechanism to ACT has become a global research goal. The selective pressure of ACT and the possibility that some specific *Pfcrt* and *Pfmdr1* alleles are associated with treatment failures was assessed in a clinical trial comparing ASAQ to AL in Nanoro. Dried blood spots collected on Day 0 and on the day of recurrent parasitaemia during the 28-day follow-up were analyzed using the restriction fragments length polymorphism (PCR-RFLP) method to detect single nucleotide polymorphisms (*SNPs*) in *Pfcrt* (codon76) and *Pfmdr1* (codons 86, 184, 1034, 1042, and 1246) genes. Multivariate analysis of the relationship between the presence of *Pfcrt* and *Pfmdr1* alleles and treatment outcome was performed. AL and ASAQ exerted opposite trends in selecting *Pfcrt K76T* and *Pfmdr1-N86Y* alleles, raising the potential beneficial effect of using diverse ACT at the same time as first line treatments to reduce the selective pressure by each treatment regimen. No clear association between the presence of *Pfcrt* and *Pfmdr1* alleles carried at baseline and treatment failure was observed.

## Introduction

*Plasmodium falciparum* resistance to antimalarial drugs is the main challenge for malaria control in endemic countries. To overcome resistance to chloroquine (CQ), artemisinin-based combination therapies (ACT) have been recommended by the World Health Organization (WHO) for the treatment of uncomplicated malaria [1]. To date, ACT has been adopted in up to 84 countries as first line treatment for uncomplicated malaria. In Burkina Faso, Artesunate-Amodiaquine (ASAQ) and Artemether–Lumefantrine (AL) are now the two first line malaria regimens in replacement of CQ (when CQ resistance reached its critical level in the country) [2] and a good efficacy and safety of these drugs is hitherto recorded [3, 4]. However recently, parasite resistance to artemisinin has been reported in five South-East Asian countries: in Cambodia, the Lao People’s Democratic Republic, Myanmar, Thailand and Viet Nam [5–7]. Moreover a decrease of ACT sensitivity has also been recorded in diverse studies including those carried out in sub-Saharan Africa [8, 9]. Due to a lack of an alternative antimalarial medicine with the same level of efficacy and tolerability as ACT at present, some effort should be deployed to determine at an early stage tools that can be used to monitor the rise and spread of artemisinin resistance in sub-Saharan Africa. For this reason, the need for a valid molecular markers associated with *P*. *falciparum* resistance to ACT is a major concern since it could allow for a more precise mapping and monitoring of the spread of resistance. The Kelch-13 marker has been associated with artemisinin resistance *in vivo and vitro* although its assessment is quite laborious for most of the sub-Saharan Africa countries [10]. Single nucleotide polymorphisms (*SNPs*) in *P*. *falciparum* CQ resistance transporter gene (*Pfcrt*) and *P*. *falciparum* multidrug resistance gene 1 (*Pfmdr1*) have been known to be associated with aminoquinoline resistance [11]. We explored the possible involvement of those markers in *P*. *falciparum* treatment failures to ACT [12, 13]. In addition, resistance could occur as a consequence of a selective pressure of an antimalarial regimen leading to a disappearance of sensitive strains and a proliferation of resistant strains. Therefore the selective impact of ACT needs to be closely monitored following their ongoing large scale deployment at community level. However since their adoption in Burkina Faso, few data on the selective impact of ACT in the circulating parasite population is available. It is in the light of all these aspects that this study was carried out with the aim of assessing the selective impact of the treatment with ASAQ and AL for *Pfcrt* and *Pfmdr1* alleles and to correlate the presence of those alleles with *in vivo* treatment failures in Nanoro, Burkina Faso.

## Material and Methods

### Study area

The study was carried out at two peripheral health facilities (Nanoro and Nazoanga) of the Nanoro Health District (NHD) situated in the central part of Burkina Faso. Nanoro is located in the Sudanese savannah zone with two distinct seasons: a rainy season occurring from June /July to October/November followed by a long dry season from November to May. Malaria transmission is highest in the rainy season with a peak located around October-November. *P*. *falciparum* is the most prevalent species. Recent entomological data reported in the country indicates that *An*. *gambiae s*.*s*. and *An*. *arabiensis* are the main vectors for malaria transmission [14]. NHD encompasses an area of 1302 km^2^ with an approximate population of 158.127 inhabitants in 2014 [15]. Population is composed of three major ethnic groups: Mossi, Gourounssi and Fulani and the majority of the population practice subsistence farming [16].

### Source of samples and study design

Samples analyzed in this study have been collected from a pharmacovigilance study whose one component aimed at assessing the effectiveness of ASAQ versus AL with a molecular analysis of resistance markers nested to it. Details of the study methodology have been described elsewhere (ClinicalTrials.gov Identifier: NCT01232530). Briefly, patients suffering of uncomplicated falciparum malaria were recruited and randomly assigned to receive either AL or ASAQ and were followed up for 28 days with scheduled visits on day 3, 7, 14, 21, and 28. At each visit, blood samples were collected for microscopic examination, haemoglobin measurement and spotted onto filter paper (Whatman 3MM, Maidstone, UK) for later PCR analyses. Details of the effectiveness trial results have been reported elsewhere [17]. For the current analysis; all dried blood spots from day 0 (before treatment) and at the day of recurrent parasitaemia (recrudescence + new infection) during the follow-up were genotyped to detect *SNPs* in *Pfmdr1* and *Pfcrt* (codon76) genes.

### DNA isolation

DNA isolation was performed at the molecular Biology laboratory of Centre Muraz located at Bobo-Dioulasso, Burkina Faso. *P*. *falciparum* DNA was extracted from dried blood spots using QIamp DNA miniKit (Qiagen, Germany) following the manufacturers procedures and 80μL of DNA template was obtained. DNA was either used immediately for a polymerase chain reaction (PCR) or stored at -20°C for later PCR analyses.

### Distinction between recrudescence and new infection

Nested PCR approach was used to assess polymorphism in two polymorphic loci (merozoite surface proteins *msp1* and *msp2*) in order to distinguish between recrudescence and new infections as previously described elsewhere [18]. Briefly, DNA fragments obtained from amplification of baseline sample (day 0) and on the day of recurrent parasitaemia were compared according to band size and number, considering the 3 families of *msp1* (Mad20, RO33, K1) and the 2 families of *msp2* (3D7, FC27). Cases were categorized as recrudescence when there was at least one common band between baseline sample and that of the day of parasite reappearance for either the 2 markers (even if there were additional bands on day 0). However, when there were no common bands between day 0 and the day of recurrent parasitaemia, patient was categorized as new infection. Cases were considered not to be clinical failures if their recurrent parasitaemia was classified as new infection rather than recrudescent infection.

### Detection of *SNPs* in *Pfcrt* and *Pfmdr1* genes

Detection of *SNPs* in *Pfcrt and Pfmdr1* genes was performed using nested PCR method followed by a restriction fragment length polymorphism (RFLP) as described by Dorsey et al [19]. Briefly, an initial amplification of the outer region of each gene was followed by nested PCR using specific primers. For *Pfcrt* the restriction enzyme *Apo I* was used for the digestion of PCR products. For *Pfmdr1* the following restriction enzymes were used: *Afl III* (NEB), *Dra I* (NEB), *Dde I* (NEB), *Ase I* (NEB), and *Eco R V* (NEB) respectively for *Pfmdr1-N86Y*, *Pfmdr1*-Y184F, *Pfmdr1*-S1034C, *Pfmdr1*- N1042D and, *Pfmdr1*-D1246Y. After digestion, DNA bands were visualized in ethidium bromide-stained 2.5% agarose gels for 2 hours at 80 V. 3D7 were used as wild type control for both *Pfcrt* (codon 76) and *Pfmdr1* and the following mutant controls were used: *Dd2* (*Pfmdr1*-N86Y), *7G8* (*Pfmdr1* Y184F/S1034C/N1042D/D1246Y, *Pfcrt*-K76T).

### Statistical analysis

Clinical Data were double entered in an ACCESS database by two independent data clerks. Molecular data were entered independently from treatment outcomes in an Excel database. Statistical analysis was performed using STATA (IC), version 10.0 software. *Pfcrt* and *Pfmdr1* genotype profile was determined by the presence or absence of wild/mutant alleles. Samples carrying both wild and mutant *Pfcrt or Pfmdr1* alleles and for which related frequencies could not be determined were excluded from the analysis. Differences between groups were assessed using the Chi-square test for proportions and a P-value of less than 0.05 was considered as statistically significant. A logistic regression was used to detect mutations that were predictive of AL and ASAQ treatment failure.

### Ethical consideration

This was part of a larger study entitled ‘‘Pharmacovigilance for artemisinin-based combination treatments in Africa (ClinicalTrials.gov Identifier: NCT01232530). A signed informed consent was obtained from each participant before enrolment. The study was reviewed and approved by the Institutional Ethics Committee of Muraz center, the National Ethics Committee of Burkina Faso, and the Ethical Review Committee of the World Health Organization (WHO).

## Results

### Clinical and parasitological responses

The clinical and parasitological responses of both AL and ASAQ treatment have been reported elsewhere [17]. Briefly, a total of 680 patients with uncomplicated malaria were randomized to receive either ASAQ (n = 340) or AL (n = 340). PCR uncorrected parasitological failure rate of 52.2% and 43.0% were reported for AL and ASAQ treatment groups respectively. By 28 days follow-up, *msp1 and msp2* genotyping analysis showed that recrudescent infections concerned 121 patients (n = 74 in AL group and n = 52 in ASAQ group), 156 patients were infected by new *P*. *falciparum* strains (n = 100 in AL group and n = 56 in ASAQ group).

### Baseline Prevalence of *Pfcrt* and *Pfmdr1* alleles by treatment group

**Fig 1:**
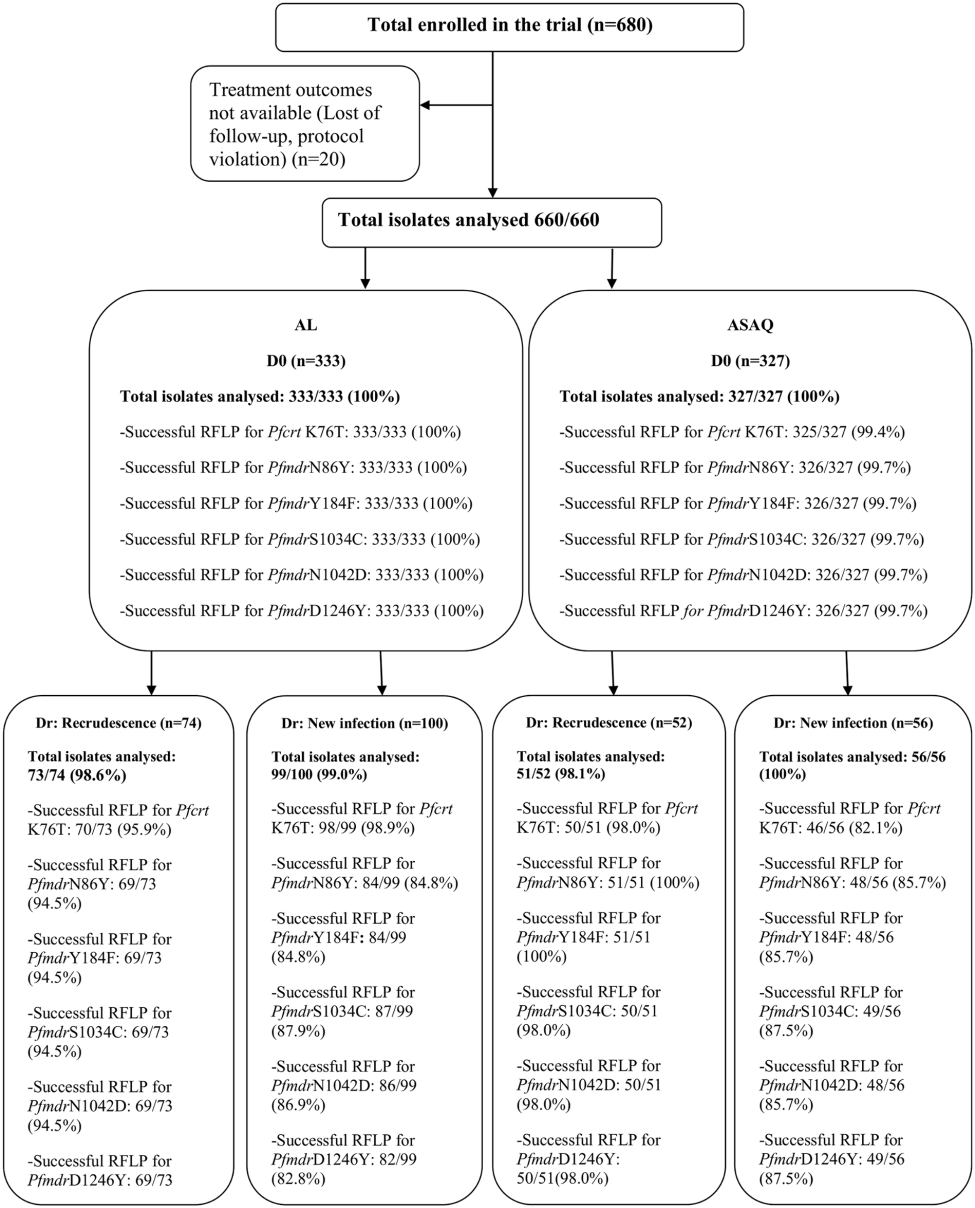
Trial profile. The figure shows the number of isolates analyzed at day 0 and on the Day of recurrent parasitaemia (recrudescence and new infections) by treatment group (AL and ASAQ) and the number of successful PCR-RFLP amplification.

Prior to treatment (Day 0), *SNPs* in *Pfcrt-K76T* and *Pfmdr1* (*N86Y*, *Y184F*, *S1034C*, *N1042D*, *D1246Y*) genes were systematically determined in all samples whose treatment outcomes were available (n = 660), stratified by treatment group. Fig 1 shows the trial profile indicating the number of isolates analyzed by treatment group and the number of successful PCR-RFLP amplification (Fig 1). At baseline, prevalence of *Pfcrt*-K76T mutation was almost similar in both AL and ASAQ treatment group (21.80% versus 20.21% respectively). Prevalence of *Pfmdr1*-N86Y mutation was also similar in the two treatment groups (8.31% for AL versus 8.25% for ASAQ). Considering *Pfmdr1*-Y184F, its prevalence was higher in ASAQ group than in AL group but the difference was not statistically significant (53.26% versus 49.82%). Extremely few cases of mutation in *Pfmdr1*-D1246Y were found (n = 3). No sample showed mutation at *Pfmdr1*-S1034C and *Pfmdr1*-N1042D loci in both AL and ASAQ treatment groups.

### Impact of AL and ASAQ treatment in selecting *Pfcrt* and *Pfmdr1* alleles

The selective impact of AL and ASAQ treatment for particular *Pfcrt* and *Pfmdr1* alleles was assessed by comparing on one side prevalence between baseline and in recrudescent infections group and on the other side between baseline and in patient with newly acquired infections within 28 days after treatment. Table 1 shows the prevalence of *Pfcrt* and *Pfmdr1* alleles before and after AL treatment.

**Table 1:** Prevalence of *Pfcrt* and *Pfmdr1* alleles before and after treatment with AL % (n/N).

Gene	Genotype profile	Pre-Treatment	Recrudescence	P-value	New infection	P-value
*Pfcrt*	K76	78.20 (226/289)	93.33 (56/60)	<0.00001	87.95 (73/83)	0.0487
	76T	21.80 (63/289)	6.67 (4/60)		12.05 (10/83)	
*Pfmdr1*	N86	91.69 (276/301)	95.65 (66/69)	0.2621	95.18 (79/83)	0.2868
	86Y	8.31 (25/301)	4.35 (3/69)		4.82 (4/83)	
*Pfmdr1*	Y184	50.18 (140/279)	55.74 (34/61)	0.4313	41.25 (33/80)	0.1588
	184F	49.82 (139/279)	44.26 (27/61)		58.75 (47/80)	

The wild *Pfcrt*-K76 allele increased significantly after treatment with AL in both recrudescence (p<0.00001) and new infection groups (p = 0.0487) while a decrease of the mutant *Pfcrt* T76 allele was observed. There was an increase of the wild N86 allele after treatment with AL in the two groups (recrudescence and new infection) but the difference was not statistically significant. Table 2 shows the prevalence of *Pfcrt* and *Pfmdr1* alleles before and after ASAQ treatment.

**Table 2:** Prevalence of *Pfcrt* and *Pfmdr1* alleles before and after treatment with ASAQ % (n/N).

Gene	Genotype profile	Pre-Treatment	Recrudescence	P-value	New infection	P-value
*Pfcrt*	K76	79.79 (225/282)	57.89 (22/38)	0.0025	34.21 (13/38)	<0.00001
	76T	20.21 (57/282)	42.11 (16/38)		65.79 (25/38)	
*Pfmdr1*	N86	91.75 (267/291)	89.36 (42/47)	0.5873	73.81 (31/42)	0.0004
	86Y	8.25 (24/291)	10.64 (5/47)		26.19 (11/42)	
*Pfmdr1*	Y184	46.74 (136/291)	39.13 (18/46)	0.3356	40.43 (19/47)	0.4205
	184F	53.26 (155/291)	60.87(28/46)		59.57 (28/47)	

Unlike AL, the mutant *Pfcrt* 76T allele increased significantly after treatment with ASAQ in both recrudescence (p = 0.0025) and new infection (p<0.00001) groups while a decrease of the wild *Pfcrt* K76 was observed. After treatment with ASAQ there was also an increase of the mutant 86Y and the difference was statistically significant in the new infection group (p = 0.0004). No evidence of selection of *Pfmdr1*-Y184F alleles after treatment with ASAQ was observed.

### Relationship between *SNPs* in *Pfcrt* and *Pfmdr1* genes with treatment outcomes

Multivariate analysis of the relationship between the presence of *Pfcrt* and *Pfmdr1* alleles and treatment outcome was performed. The effect of age on this association was evaluated after stratification of the patients into two groups (<5 years and ≥5 years) regarding the significant importance of this distinction in malaria epidemiology. Haemoglobin (Hb) level and parasite density (PD) were also stratified into two groups (<Hb/PD median and ≥Hb/PD median). Table 3 shows results of the multivariate analysis of the association between the presence of *Pfcrt* and *Pfmdr1* alleles and AL treatment failures.

**Table 3:** Multivariate analysis of the association between *SNPs* in *Pfcrt* and *Pfmdr1* genes and AL treatment failure.

	Crude OR	95%CI	P-value	Adjusted OR	95%CI	P-value
***Age group***						
*< 5 years*	1.43	0.74–2.73	0.277			
*≥ 5 years*	1.00	-	-			
***Haemoglobin level***						
***<****median = 9 g/dl*	1.25	0.73–2.16	0.404			
***≥*** *median = 9 g/dl*	1.00	-	-			
***Parasite density***						
***<*** *G median = 30529 /μl*	1.00	-	-			
***≥*** *G median = 30529 /μl*	1.16	0.68–1.98	0.566			
***Pfcrt K76T***						
*Wild*	1.70	0.78–3.69	0.176	1.15	0.47–2.81	0.751
*Mutant*	1.00	-	-	1.00	-	-
***Pfmdr1 N86Y***						
*Wild*	1.00	-	-	1.00	-	-
*Mutant*	1.16	0.44–3.03	0.760	2.59	0.82–8.08	0.101
***Pfmdr1-Y184F***						
*Wild*	1.00	-	-	1.00	-	-
*Mutant*	0.46	0.25–0.85	0.014	0.37	0.16–0.83	0.017

A significant association with AL treatment failure was found, with the presence on day 0 of *Pfmdr1*-Y184 allele but not with others *Pfmdr1*and *Pfcrt* alleles.

Table 4 shows results of the multivariate analysis of the association between the presence of *Pfcrt* and *Pfmdr1* alleles and ASAQ treatment failures.

**Table 4:** Multivariate analysis of the association between *SNPs* in *Pfcrt* and *Pfmdr1* genes and ASAQ treatment failure.

	Crude OR	95%CI	P-value	Adjusted OR	95%CI	P-value
***Age group***						
*< 5 years*	2.25	0.97–5.24	0.058			
*≥ 5 years*	1.00	-	-			
***Haemoglobin level***						
***<****median = 9 g/dl*	1.00	-	-			-
***≥*** *median = 9g/dl*	0.46	0.25–0.86	0.016			
***Parasite density***						
***<*** *median = 30762 /μl*	1.00	-	-			-
***≥*** *median = 30762 /μl*	1.46	0.78–2.73	0.225			
***Pfcrt K76T***						
*Wild*	1.00	-	-	1.00	-	-
*Mutant*	1.20	0.55–2.61	0.633	1.68	0.67–4.21	0.263
***Pfmdr1 N86Y***						
*Wild*	1.00	-	-	1.00	-	-
*Mutant*	0.86	0.24–3.02	0.815	0.78	0.20–3.01	0.729
***Pfmdr1-Y184F***						
*Wild*	1.00	-	-	1.00	-	-
*Mutant*	0.95	0.49–1.84	0.901	0.96	0.43–2.14	0.932

No association was observed between the presence of *Pfcrt* and *Pfmdr1* alleles carried at baseline and ASAQ treatment failure before and after adjustment according to multivariate analysis.

## Discussion

The understanding of *P*. *falciparum* resistance mechanisms to ACT is now a major concern since a decrease of their sensitivity and some cases of resistance have been reported in some part of the world. In this study we report on the selective pressure of the two recommended treatments for uncomplicated malaria in Burkina Faso and on the correlation between *SNPs* in *Pfcrt* and *Pfmdr1* genes and *in vivo* treatment failure.

We found a similar baseline prevalence of *Pfcrt* and *Pfmdr1* alleles between the two treatment groups. This similarity of baseline prevalence in the two arms may indicate that any significant change of prevalence in post-treatment was drug dependent. Furthermore, except for mutation *Pfmdr1*-Y184F, a lower baseline prevalence of mutation in the two genes was found. Indeed, Burkina Faso was a setting of high CQ resistance (with extremely high prevalence of those mutations) [2] and this lowering of prevalence is attributable to the change of treatment policy from CQ to ACT since 2005 [20]. The high prevalence of *Pfmdr1*-Y184F mutation in the country was previously recorded [21].

Our findings show positive selection of the Wild *Pfcrt*-K76 and *Pfmdr1*-N86 alleles after treatment with AL which corroborates previous reports in several settings [22–25]. Interestingly, this does not affect the efficacy of this treatment regimen because the selecting strains were not associated with treatment failure. However, in a recent study carried out by Venkatesan et al *Pfmdr1*-N86 allele was identified as an independent risk factor for recrudescence in patient treated with AL meaning that this selective pressure needs to be closely monitored [26].

We also found a positive selection of the mutant *Pfcrt*-76T and *Pfmdr*1-86Y alleles after treatment with ASAQ. We postulate that this positive selection of mutant type by ASAQ is due to the presence of AQ (structurally similar to CQ) as partner drug in this combination [27, 28]. In this regard, though the selected alleles were not associated with treatment failure in the present study, ASAQ efficacy could be affected in the future (when the selected resistant strains will be predominant in the area) by decreasing sensitivity to AQ in an area of high CQ resistance (few years before) such as Burkina Faso. This means that in such context, AL might be more appropriate in setting of high CQ resistance (with high prevalence of mutant allele) than ASAQ.

The observations stated above show obviously that AL and ASAQ exerted opposite trend in term of selecting parasite strains. In Burkina Faso, the two drugs have been adopted at the same time as first line treatments of uncomplicated malaria. Thus, these findings raise the beneficial effect of using this strategy in term of avoiding the selection of resistant circulating *P*. *falciparum* strains by one treatment regimen.

Our findings suggest also no association between the presence of *Pfcrt* and *Pfmdr1* alleles carried at baseline and ASAQ treatment failure, consistent with a previous report [26]. The *Pfmdr1*-Y184 allele appeared to be associated with AL treatment failure. However, this mutation was not under significant selection pressure after AL treatment. Moreover, previous work has described an association of the Y184F mutation with decreasing sensitivity to lumefantrine [29]. Therefore, the observation might be simply a chance finding. The relationship between the carriage of *Pfcrt* and *Pfmdr1* alleles and AL and ASAQ treatment outcome would have been easier to assess if drug intake was supervised in the effectiveness study. This was mentioned as a limit of our study in the previous report [17]. Furthermore, the use of two markers (*msp1* and *msp2*) instead of three [*msp1*, *msp2*, and *glurp* (*glutamate rich protein*)] to distinguish between recrudescence and new infections constitutes another limit of this study and could partially explain why no significant difference was observed between the two groups (recrudescence and new infections). In this study the prevalence of mutation in *Pfmdr1*-D1246Y was extremely low and it was difficult to correlate the presence of this mutation and treatment outcomes. No samples carried mutation in *Pfmdr1*-S1034C and *Pfmdr1*-N1042D as reported by several studies throughout Africa [21, 30].

## Conclusion

AL and ASAQ exerted opposite trends in selecting *Pfcrt K76T* and *Pfmdr1-N86Y* alleles, raising the potential beneficial effect of using diverse ACT at the same time as first line treatments to reduce the selecting pressure by each treatment regimen. No association between the presence of *Pfcrt* and *Pfmdr1* alleles carried at baseline and ASAQ treatment failure was observed.

## Supporting Information

S1 Fig Trial profile showing the number of samples analysed at day 0 and among recrudescence and new infections by treatment arm. (XLSX)
